# Impact of Technology on Neonatal Intensive Care Unit Admissions and Length of Stay: A Retrospective Study

**DOI:** 10.7759/cureus.40813

**Published:** 2023-06-22

**Authors:** Lisa B Shields, Yevgeniy Davydov, Angela Glyder, Clayton Weymouth, Michael Udwin, Matt Eakins

**Affiliations:** 1 Norton Neuroscience Institute, Norton Healthcare, Louisville, USA; 2 Data Analytics, Lucina, Boca Raton, USA; 3 Clinical Operations, Lucina, Boca Raton, USA; 4 Obstetrics and Gynecology, CareFirst BlueCross BlueShield, Baltimore, USA

**Keywords:** public health, perinatology, pediatrics, health technology, health services delivery, length of stay, neonatal intensive care units, obstetrics, pregnancy

## Abstract

Background

Neonatal intensive care units (NICU) provide essential medical care to neonates; however, they are associated with hospital-acquired infections, less maternal-newborn bonding, and high costs. Implementing strategies to lower NICU admission rates and shorten NICU length of stay (LOS) is essential. This study uses causal-inference methods to evaluate the impact of care managers using new technology to identify and risk stratify pregnancies on NICU admissions and NICU LOS. The NICU LOS will decrease as a result of the use of new technology by care managers.

Study design

This retrospective study utilized delivery claims data of pregnant women from the CareFirst BlueCross BlueShield Community Health Plan District of Columbia from 2013 to 2022, which includes the pre-intervention period before the use of new technology by care managers and the post-intervention period with the use of new technology by care managers. Our sample had 4,917 deliveries whose maternal comorbidities were matched with their neonate’s outcomes.

Methods

To evaluate the impact of the technological intervention, both Generalized Linear Models (GLMs) and Bayesian Structural Time-Series (BSTS) models were used.

Results

Our findings from the GLM models suggest an overall average reduction in the odds of NICU admissions of 29.2% and an average decrease in NICU LOS from 7.5%-58.5%. Using BSTS models, we estimate counterfactuals for NICU admissions and NICU LOS, which suggest an average reduction in 48 NICU admissions and 528 NICU days per year.

Conclusion

Equipping care managers with better technological tools can lead to significant improvements in neonatal health outcomes as indicated by a reduction in NICU admissions and NICU LOS.

## Introduction

Approximately 10-15% of infants born in the United States are admitted to the neonatal intensive care unit (NICU) annually [1]. While NICUs offer life-saving measures for neonates with improved survival, they are also associated with significant morbidity and mortality, high healthcare costs, and increased risk of nosocomial infections [2-8]. Numerous risk factors are correlated with increased odds of NICU admissions, including prematurity, maternal diabetes mellitus, hypertension, premature rupture of membranes (PROM), C-section, neonatal abstinence syndrome, and multiple gestations [2,9-12]. Several risk factors are associated with a neonate’s increased length of stay (LOS) in the NICU, including ventilator-dependent, low birth weight, low 5-minute Apgar score, surfactant administration, and infections [6,7,13,14].

We developed a new technology based on a real-time scoring algorithm to identify and risk-stratify pregnancies based on various risk factors that can lead to poor birth outcomes [15]. With this new technology, claims data are mined daily for new pregnancies, and continuous updates are provided to existing pregnancies. Identification and risk stratification of pregnancies, especially in the first trimester, allows for more efficient and timely care management.

Identifying women early in their pregnancy allows care managers to provide resources that optimize the health of pregnant women, which subsequently improves birth outcomes. CareFirst BlueCross BlueShield Community Health Plan District of Columbia (CHPDC) is a for-profit healthcare company that offers health insurance benefits and services to over 66,000 Medicaid enrollees in the District of Columbia [16]. CareFirst CHPDC started using the new technology on July 30, 2019. This new technology provides data analytics tools that care managers at CareFirst CHPDC previously did not have, including pregnancy identification, risk stratification, and other analytic solutions such as dashboards for care managers to better track and manage enrollees throughout their pregnancy.

The goal of the present study was to determine the impact of the new technology on NICU admissions and LOS for CareFirst CHPDC. We investigated whether the new technology intervention was associated with a decrease in the odds of NICU admissions and LOS. The uniqueness of this study is twofold: 1) We used delivery claims data from a large Medicaid population over a seven-and-a-half (7½) year period from January 2013 to May 2022 to perform a program evaluation of the use of new technology, which identified and risk-stratified pregnancies by care managers on NICU outcomes and 2) Both traditional generalized linear models (GLM) (Logit, Poisson) were used as well as Bayesian-Structural Time-Series (BSTS) models to achieve robust estimates of the impact of the new technology on NICU outcomes.

## Materials and methods

Study population

Under an Institutional Review Board (IRB)-approved protocol and according to the Declaration of Helsinki, a dataset was created using data from CareFirst CHPDC over a seven-and-a-half (7½) year period from January 2013 to May 2022. The population of 4,917 deliveries were those deliveries from CareFirst CHPDC where maternal data was matched with neonatal data. This allowed us to control for maternal comorbidities in order to estimate the true effect of the new technology on neonatal outcomes. We evaluated the pre-intervention (before the use of new technology by care managers) period (January 2013-November 2019) compared to the post-intervention period (December 2019-May 2022) to determine the influence of the new technology on CareFirst CHPDC. All multifetal and preterm births were included and controlled for in the models. Demographic, neonatal, and obstetric characteristics were analyzed and controlled for in the models.

**Figure 1:**
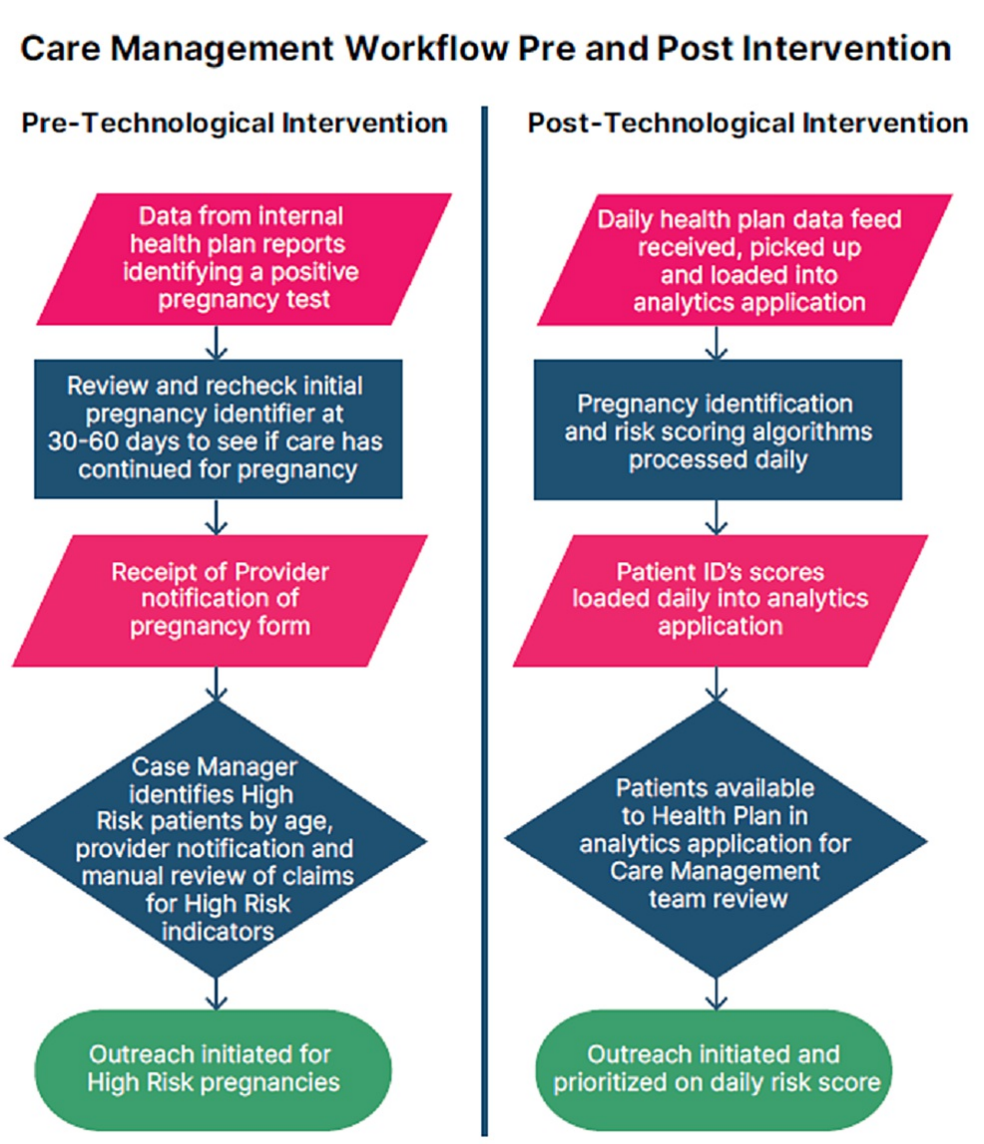
Care Management Workflow Care management workflow depicting the pre- and post-technological interventions

The “technological intervention” refers to the use of data analytics tools to identify and risk-stratify pregnancies by CareFirst CHPDC care managers (Figure 1).

The first step in the intervention was for data from all CareFirst CHPDC enrollees to be passed into the new technology. The data were subsequently mined for pregnancies, and risk stratification of new and existing pregnancies was performed. This resulted in a new web-based application that focused on providing identification and continuous risk stratification of pregnant women. Additionally, this technology provided prioritization tools that enabled the care manager to drill into specific cross-sections in their pregnant population. For example, a care manager could use the technology to find enrollees who fit the following criteria: are less than 14 weeks of gestation, have a history of preterm birth, and have a history of drug use. Once they have a list of these enrollees, the risk score aided the care manager by creating a ranking of those enrollees who met the filter criteria. The care manager was able to outreach by focusing on the enrollees with the highest scores first. This new technology was deployed to the CareFirst CHPDC market on July 30, 2019. The goal was to provide daily risk stratification and continuous reporting on the maternal and neonatal populations. The Western IRB-Copernicus Group (WCG) IRB determined that this study was exempt under 45 CFR 46.104(d)(4).

Data collection and variables

Data were obtained from CareFirst CHPDC’s electronic health claim records. Maternal data included age, socioeconomic status, zip code, and maternal comorbidities such as sickle cell disease, pre-existing diabetes mellitus, and hypertension (Table 1).

**Table 1:** Neonatal Intensive Care Unit Admissions and Length of Stay – Descriptive Statistics NICU: neonatal intensive care unit

	NICU Admissions			NICU Length of Stay (Days)		
Characteristic	N	0, N = 3,578	1, N = 1,339	N	Mean	Standard Deviation
Technological intervention	4,917	1,148 (32%)	483 (36%)	4,917	8.65	14.92
Multifetal pregnancy	4,917	44 (1.2%)	44 (3.3%)	4,917	17.43	16.4
Prior multifetal pregnancy	4,917	9 (0.3%)	6 (0.4%)	4,917	6	5.14
Condition complicating pregnancy	4,917	1,863 (52%)	964 (72%)	4,917	8.27	18.39
Anemia	4,917	888 (25%)	568 (42%)	4,917	6.56	11.26
High-risk pregnancy	4,917	1,804 (50%)	964 (72%)	4,917	8.26	18.34
Pre-existing hypertension	4,917	272 (7.6%)	183 (14%)	4,917	13.13	32.68
Gestational diabetes mellitus	4,917	449 (13%)	194 (14%)	4,917	8.19	14.53
Prior miscarriage	4,917	101 (2.8%)	73 (5.5%)	4,917	8.32	14.53
Advanced maternal age (≥ 35 years)	4,917	554 (15%)	191 (14%)	4,917	10.22	16.76
Gestational hypertension	4,917	279 (7.8%)	200 (15%)	4,917	7.82	12.54
Obesity/high body mass index	4,917	910 (25%)	454 (34%)	4,917	7.37	12.35
Drug use	4,917	203 (5.7%)	230 (17%)	4,917	11.32	28.84
Tobacco/nicotine use	4,917	305 (8.5%)	249 (19%)	4,917	9.89	26.8
History of premature rupture of membranes	4,917	143 (4.0%)	125 (9.3%)	4,917	14.38	18.37
Uterine/cervix anomalies	4,917	670 (19%)	396 (30%)	4,917	6.89	12.57
Hemorrhage	4,917	520 (15%)	347 (26%)	4,917	9.05	23.9
Young pregnancy (≤ 18 years)	4,917	109 (3.0%)	66 (4.9%)	4,917	7.08	14.14
Insufficient prenatal care	4,917	641 (18%)	396 (30%)	4,917	8.34	23.6
Behavioral health condition	4,917	245 (6.8%)	192 (14%)	4,917	10.74	30.38
Depression	4,917	204 (5.7%)	145 (11%)	4,917	8.06	12.31
Thrombophilias	4,917	99 (2.8%)	61 (4.6%)	4,917	6.72	9.79
Preterm labor without delivery	4,917	242 (6.8%)	244 (18%)	4,917	7.89	11.83
History of preterm labor	4,917	172 (4.8%)	138 (10%)	4,917	6.8	9.37
Group B streptococcal infection	4,917	324 (9.1%)	194 (14%)	4,917	6.66	11.52
Nutritional deficiency	4,917	94 (2.6%)	44 (3.3%)	4,917	10.66	20.11
Sexually transmitted disease	4,917	678 (19%)	457 (34%)	4,917	7.27	12.93
Pre-existing diabetes mellitus	4,917	90 (2.5%)	82 (6.1%)	4,917	12.02	19.34
History of post-term delivery	4,917	304 (8.5%)	173 (13%)	4,917	4.03	5.89
Chronic renal disease	4,917	22 (0.6%)	17 (1.3%)	4,917	20	26.21
Low socioeconomic status	4,917	126 (3.5%)	38 (2.8%)	4,917	7.97	11.49
Infections (non-sexually transmitted diseases)	4,917	243 (6.8%)	160 (12%)	4,917	7.98	13.37
Anxiety	4,917	161 (4.5%)	93 (6.9%)	4,917	6.53	9.04
Fetal abnormality	4,917	420 (12%)	239 (18%)	4,917	9.49	16.35
Spotting	4,917	154 (4.3%)	83 (6.2%)	4,917	6.67	10.5
Unwanted pregnancy	4,917	7 (0.2%)	3 (0.2%)	4,917	55.33	85.54
Placenta previa	4,917	37 (1.0%)	31 (2.3%)	4,917	9.9	12.93
Non-gynecological cancer	4,917	7 (0.2%)	7 (0.5%)	4,917	5.43	6.13
Genitourinary infections	4,917	656 (18%)	386 (29%)	4,917	8.09	13.5
Physical/emotional abuse	4,917	36 (1.0%)	26 (1.9%)	4,917	7.31	10.75
Cardiac disease and complications	4,917	56 (1.6%)	45 (3.4%)	4,917	6.18	7.26
Thyroid disease	4,917	64 (1.8%)	41 (3.1%)	4,917	12.98	21.78
Gingivitis/periodontal disease	4,917	4 (0.1%)	6 (0.4%)	4,917	3.67	2.73
Alcohol use	4,917	37 (1.0%)	40 (3.0%)	4,917	7.05	9.08
Cervical shortening	4,917	46 (1.3%)	33 (2.5%)	4,917	18.3	21.14
Cervical incompetence	4,917	52 (1.5%)	41 (3.1%)	4,917	12.88	15.65
Intrauterine growth restriction (IUGR)	4,917	187 (5.2%)	124 (9.3%)	4,917	12.4	17.94
Cerclage procedure	4,917	22 (0.6%)	14 (1.0%)	4,917	17.57	16.55
Pre-eclampsia	4,917	145 (4.1%)	132 (9.9%)	4,917	13.96	36.63
Homelessness	4,917	24 (0.7%)	14 (1.0%)	4,917	10.29	10.44
Domestic violence	4,917	22 (0.6%)	15 (1.1%)	4,917	5.93	10.48
Low weight gain during pregnancy	4,917	24 (0.7%)	14 (1.0%)	4,917	8	9.58
Sleep disorder	4,917	30 (0.8%)	20 (1.5%)	4,917	9.35	9.81
Gynecological cancer	4,917	1 (<0.1%)	4 (0.3%)	4,917	6.25	5.91
Lupus	4,917	7 (0.2%)	3 (0.2%)	4,917	2	1.73
History of preterm delivery	4,917	20 (0.6%)	34 (2.5%)	4,917	19.76	66.2
Eating disorder	4,917	13 (0.4%)	4 (0.3%)	4,917	2.75	0.5
Low body mass index	4,917	5 (0.1%)	3 (0.2%)	4,917	3	1
Fetal fibronectin procedure	4,917	7 (0.2%)	3 (0.2%)	4,917	14.67	11.68
Endometriosis	4,917	10 (0.3%)	5 (0.4%)	4,917	6	7.84
Arteriosclerosis	4,917	2 (<0.1%)	2 (0.1%)	4,917	14	12.73
Uterine overdistension	4,917	130 (3.6%)	63 (4.7%)	4,917	8.94	12.57
Maternal short stature	4,917	0 (0%)	1 (<0.1%)	4,917	5	
Cervical cone	4,917	9 (0.3%)	2 (0.1%)	4,917	2	0

Obstetrical data included a history of preterm delivery, pre-eclampsia, placenta previa, fetal abnormality, gestational hypertension, C-section delivery, as well as other prior and current obstetrical conditions. The pregnant woman’s historical data were evaluated against the neonate’s NICU admissions and LOS.

The new technology model that we developed used machine learning to estimate a risk score ranging from 0 (lowest risk) - 100 (highest risk) for each patient based on all of the conditions listed in Table 1. The scores were then updated in real-time, as more information about the patient was received throughout the pregnancy. More details on the scoring model were described in our previous article [15]. Any identified pregnancy with a score greater than 70 was referred to a CareFirst CHPDC case manager. Enrollees with a score below 70 were referred to their care coordination support team for additional monitoring, screening, and referral to applicable services. However, no threshold was used in the model itself. It was our recommendation to care managers to move away from that mindset since it encouraged the "high/low-risk" binary. Instead, our suggestion was to use the tool to efficiently filter to their sub-cohort of interest. The score was used as a ranking mechanism focusing initially on enrollees who were at the highest risk.

Program evaluation

Several factors were considered after CareFirst CHPDC implemented the technological intervention, such as (1) the time it took for CareFirst CHPDC case managers to learn how to use the new technology, which included a case management application, dashboards, and analytics tools, and (2) the time it took for care managers to act on the information they received from the new technology and to contact enrollees early in their pregnancies. To account for these time considerations, the technological intervention indicator variable was adjusted by a five-month buffer period. The value of 1 was assigned for any deliveries from November 2019 and afterward, which we labeled the “technological intervention” period, and 0 for the period before, which we labeled the “pre-intervention” period. Having the technological intervention indicator variable allowed us to statistically test and evaluate the impact of the new technology on NICU admissions and LOS outcomes for CareFirst CHPDC.

Several statistical models and methods were utilized to perform the program evaluation. Classic generalized linear models (GLM) models were used for baseline results. Bayesian structural time-series (BSTS) models were used as an alternative approach to validate the program evaluation results of the GLM models. Using multiple GLM models as well as BSTS models allowed us to ensure that our results were robust to both model error and model misspecification. This allowed us to get reliable statistical estimates of the impact of the technological intervention. Statistical analyses were performed using R version 4.2.1 (R Core Team (2021). R: A language and environment for statistical computing. R Foundation for Statistical Computing, Vienna, Austria) [17].

Generalized linear models

Three enrollee-level GLM models (Logit, Poisson, and Negative Binomial) used maternal data to determine the impact of the technological intervention on NICU admissions and LOS. The GLM models were estimated with the glm() command in the stats package v. 4.2.1 [17]. To model the effect of the technological intervention on NICU admissions and LOS, the theoretical outcomes of their variables needed to be understood. The NICU admission variable took the value of 1 if there was a NICU admission in the first year of birth and the value of 0 if the neonate was not admitted to the NICU. Since both NICU admissions and LOS outcome variables were not continuous and were bounded, it was not appropriate to estimate the causal effect of the technological intervention on these outcomes using Ordinary Least Squares (OLS). We used the logit regression model to estimate the causal effect of the technological intervention on NICU admissions since it was appropriate for modeling binary choice outcomes and, in this case, NICU admissions was a binary variable. The five-month buffer was the technological intervention dummy variable that took on the value of 1 for any deliveries from December 2019 and on, and 0 for the period before. Maternal control variables, such as pre-existing diabetes mellitus and other comorbidities, were included in the models. The logit models also controlled for monthly birth volume, seasonality, and zip code. The monthly birth volume was used as a proxy for NICU utilization, and zip codes were used as a proxy for socioeconomic determinants and distance to the hospital.

The Poisson regression model was used to estimate the causal effect of the technological intervention on NICU LOS since it was appropriate for modeling count outcomes and, in this case, NICU LOS was measured in days and was an ordinal variable. One limitation of using a Poisson regression to model NICU LOS was that it is based on the Poisson distribution, which assumes that mean and variance are the same. The negative binomial distribution is a standard way to handle this limitation and to account for the excess dispersion in the data. The mean and variance of NICU LOS (days) were 8.53 and 298.57, which indicated the presence of overdispersion in our data. To ensure that our results were robust to overdispersion, we estimated the impact of the technological intervention using a negative binomial regression.

Bayesian structural time-series models

One of the challenges in the program evaluation was to infer the causal impact of an intervention on an outcome metric over time. Traditional linear models, GLM, or difference-in-difference (DD) approaches are limited in several ways, including 1) static regression models assume identically independently distributed (i.i.d.) data despite the design having a temporal component, and 2) most DD analyses only consider two time points before and after the intervention while, in practice, the effect evolves over time [18]. State-space models fill these gaps by inferring the temporal evolution of the attributable impact and accommodating multiple sources of variation such as local trends, seasonality, and time-varying influence of contemporaneous covariates [18].

The BSTS models were estimated using the bsts package v. 0.9.8 and the CausalImpact package v. 1.2.7 [18,19]. The BSTS models ensured the robustness of our GLM results and allowed us to estimate the counterfactual of not having the technological intervention in a rigorous manner. These models were applied to both the NICU admissions and LOS outcome metrics with January 2013 to November 2019 as the pre-intervention period and December 2019 to May 2022 as the post-intervention period. 

These models allowed us to obtain counterfactual predictions of NICU outcomes by constructing a synthetic control of pregnant women who delivered prior to the technological intervention. This resulted in a counterfactual time series of NICU outcomes that would be observed during the intervention period if there was no technological intervention. The impact was derived by obtaining the average difference between the actual and counterfactual NICU outcomes during the intervention period.

## Results

Claims data volume of CareFirst CHPDC

Over the seven-and-a-half (7½) year duration of this study, there were 4,917 delivery claims from neonates who were matched with maternal data from CareFirst CHPDC. This equated to an average of 656 deliveries per year. Table 1 highlights the descriptive statistics of the NICU admissions and LOS.

GLM models for NICU admissions and length of stay

The GLM models for NICU admissions and LOS are presented in Table 2.

**Table 2:** Neonatal Intensive Care Unit Admissions and Length of Stay Models NICU: neonatal intensive care unit *, **, and *** denote statistical significance at the 10%, 5%, and 1% levels. P-values are given in parentheses.

	NICU Admissions	NICU Length of Stay (Days)	
	Logit (OR)	Poisson (IRR)	Negative Binomial (IRR)
Technological Intervention	0.708***	0.925***	0.766***
	(0.001)	(0.001)	(0.006)
Maternal Comorbidities	Yes	Yes	Yes
ZIP Code Dummies	Yes	Yes	Yes
Month Dummies	Yes	Yes	Yes
N	4,917	4,917	4,917

The technological intervention for the NICU admissions was statistically significant in the Logit model with an odds ratio of 0.708, reflecting a 29.2% decrease in the odds of NICU admissions ((odds ratio-1)x100% gives the percent increase or decrease in odds). The technological intervention for NICU LOS was statistically significant in both the Poisson and negative binomial models with incidence response ratios of 0.925 and 0.766, respectively. These findings were associated with a 7.5% and 23.4% decrease in NICU days using the Poisson and negative binomial models, respectively.

BSTS model

The BSTS model for NICU admission count is shown in Table 3.

**Table 3:** Impact of Technological Intervention on Neonatal Intensive Care Unit Admissions and Length of Stay (December 2019 – May 2022) NICU: neonatal intensive care unit

Average Impact: Month/Year/%	NICU Number of Admissions (#/%)	NICU Length of Stay (Days)
Month	-4	-44
Year	-48	-528
Overall %	-19%	-24%
Probability of Causal Effect	98%	93%
Predictors used: hypertension, diabetes mellitus, pre-eclampsia, depression, endometriosis, arteriosclerosis, maternal short stature, cervical cone, preterm birth, C-section, all low birth weight, fetal abnormality, multifetal gestation, prior multifetal gestation		

**Figure 2:**
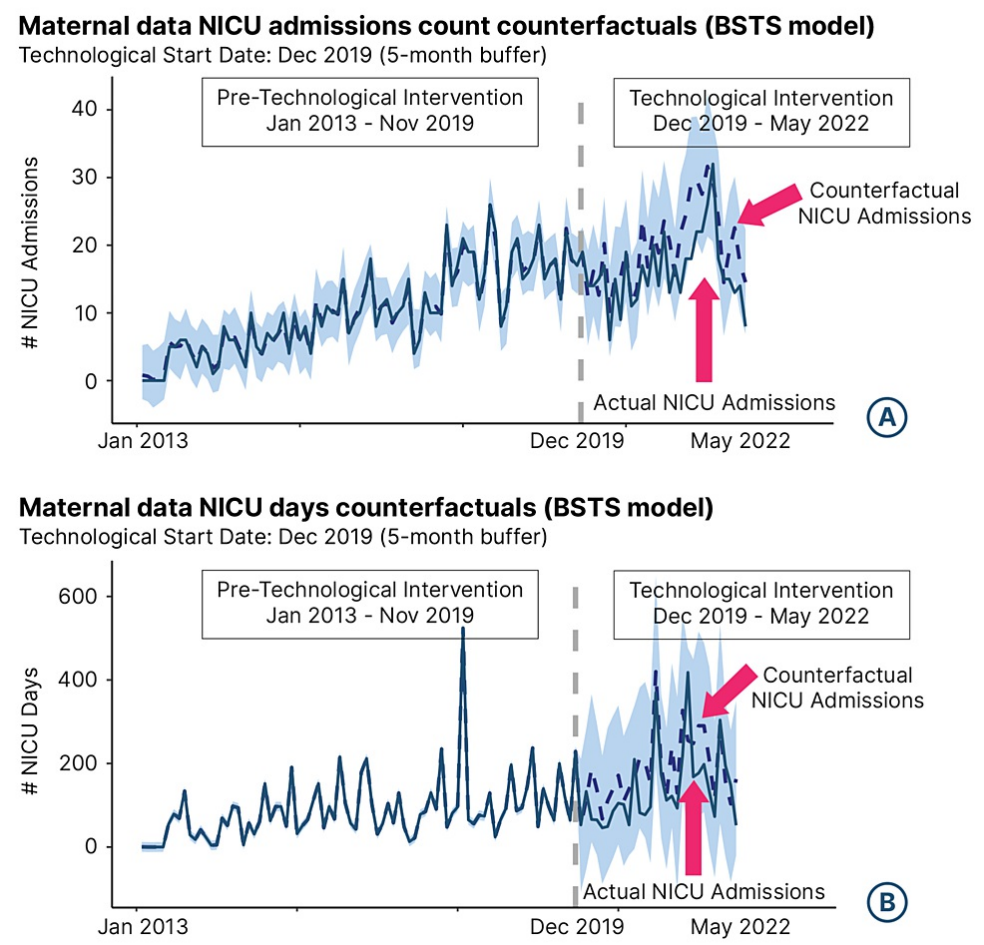
Bayesian Structural Time-Series Model Bayesian structural time-series model for (A) neonatal intensive care unit admissions count counterfactuals and (B) neonatal intensive care unit length of stay counterfactuals.

For most of the technological intervention period, the counterfactual NICU admission count was higher than the actual NICU admission count, indicating a successful intervention (Figure 2).

The difference between the actual and counterfactual NICU admission counts resulted in an average monthly reduction of four NICU admissions, which is equivalent to a reduction in 48 NICU admissions per year and an average percent reduction of 19% per month (Table 3). These findings were statistically significant with the probability of a causal effect of 98%.

The BSTS model for the number of NICU days is depicted in Figure 2 and Table 3. The counterfactual number of NICU days was higher than the actual number of NICU days for most of the technological intervention period, indicating a successful intervention (Figure 2). The difference between the actual and counterfactual NICU days resulted in an average monthly reduction of 44 NICU days, which is equivalent to an average total reduction of 528 NICUs per year and an average percentage reduction of 24% (Table 3). These results are statistically significant with the probability of a causal effect of 93%.

## Discussion

Receiving antenatal care has a profound impact on the successful outcome of neonates [20-29]. An association has been reported between late initiation of antenatal care or receiving limited antenatal visits and preterm birth or low birth weight [20]. The World Health Organization (WHO) recommends a risk-oriented approach that includes (1) routine care to all women; (2) additional care for women with moderately severe diseases and complications; and (3) specialized obstetrical and neonatal care for women with severe diseases and complications [28]. Studies have shown that a large proportion of high-risk women had a utilization or content of care below the recommended standard, which resulted in a higher number of preterm births [28,29]. Sword and colleagues reported the importance of clinical and interpersonal care processes as essential components of quality prenatal care since these processes decrease the likelihood of adverse outcomes and promote the involvement of pregnant women in their own care [26].

In Manjavidze and colleagues’ study of mothers and singleton newborns, women who received inadequate antenatal care experienced the highest odds of newborn admissions to the NICU and perinatal mortality [22]. These authors stress the importance of appropriate antenatal care before the gestational age of week 12. In Partridge and colleagues’ study of >28 million pregnant women, inadequate prenatal care utilization was more common among women ≤20 years, black non-Hispanic and Hispanic women, and those without high school education [24]. The risk of prematurity, stillbirths, early and late neonatal death, and infant death increased linearly with decreasing care.

Villar and colleagues described the findings of a WHO antenatal care randomized trial to evaluate a new model of routine antenatal care which was based on intention to treat [27]. More women in the new model were referred to higher levels of care compared to the standard model, however, routine antenatal care with the new model did not affect maternal and perinatal outcomes and may reduce cost. In Pacagnella and colleagues’ multicenter cross-sectional study of delays in receiving obstetric care and poor maternal outcomes (potentially life-threatening conditions, maternal death, or maternal near-miss cases), these authors reported a significant association between frequency of delay and severity of outcome, suggesting the vital need for timely and proper antenatal management [23].

Case management programs are associated with improved maternal and fetal outcomes in high-risk pregnancies [30]. In Chou and colleagues’ study of the effect of nurse-led antepartum case management on stress, anxiety, and pregnancy outcomes in women with pregnancy-induced hypertension, a total of 62 women were randomly assigned to an experimental group (received case management for 8 weeks) or control group (received routine clinical care) [30]. Women in the experimental group had significantly larger decreases in stress and anxiety. These authors suggested that positive effects on the psychosocial outcomes of pregnant women who received case management have valuable clinical implications [30]. Anxiety was included as a control variable in our models (Table 1). While we did not have a specific control variable for stress, “Depression”, “Behavioral Health Condition”, and “Sleep Disorder” were control variables that may be related to stress (Table 1). Since these variables were included as controls in our statistical models, our results were robust to patients experiencing stress and/or anxiety. The technological intervention was effective in improving NICU outcomes after controlling for patients with these conditions.

Increasing the access and use of antenatal care coupled with care management and technology can have positive impacts on birth outcomes. Care managers may benefit greatly from our technological advances that not only provide early identification and real-time stratification of pregnancies but also prioritize enrollees who are most in need of urgent antenatal care (Figure 1). Prior to the use of the new technology, care managers relied on multiple manual reports and/or notification forms completed by providers. The manual reports were generated from limited claims data focused on positive pregnancy tests, had limited refresh occurring every 30-60 days, and required significant time to complete and compare from one report to the next. Additionally, the manual notifications from providers were often limited in detail and completion and seen as administratively burdensome to providers. The use of the new technology application provided care managers with a way to quickly, efficiently, and accurately identify new pregnant enrollees that allowed for early outreach and interventions. This new approach also provided the care managers with the advantage of identifying more pregnant enrollees and increasing the number of enrollees they were able to engage in case management earlier in their pregnancy. The information in the application was updated daily for care manager review to facilitate more prioritized and focused outreach planning. Ensuring that pregnant enrollees were connected to early and consistent antenatal care was the priority of the care managers.

With the new technology, the additional daily risk stratification allowed the care managers to better identify and prioritize the highest-risk pregnancies. Without this new technology, the care managers were reviewing risk based solely on condition(s) that typically led to poor birth outcomes, not data-driven information that was available in real-time based on the dynamic pregnancy condition. The care manager used the application to filter risk scores and specific conditions that historically have been shown to have poor birth outcomes, potentially leading to a NICU admission and extended LOS. The flexibility of the tool provided the care manager with a way to better prioritize enrollees that needed the outreach more urgently than relying on a report that would only indicate if the pregnancy was impacted by a high-risk condition. One example of a priority outreach was a pregnant woman with a history of a previous preterm delivery and chronic hypertension. By quickly identifying the pregnancy and history, the care manager was able to prioritize the outreach and engagement of that pregnant enrollee to complete an assessment of the medical and psychosocial conditions impacting the pregnancy. The completed assessment and enrollee engagement allowed the care manager to identify specific coordination and education needs of the pregnant enrollees to develop an enrollee-centered care plan that could be shared with the enrollee and care team. Fast identification and outreach allowed both the care manager and care team more time to work with the enrollee to achieve better overall birth outcomes with consistent antenatal care and connections to community resources as needed.

Strengths and limitations

Our study evaluated the impact of technology that identifies and stratifies pregnancies early in gestation on NICU admissions and LOS. The current study boasts several strengths, including two separate models of NICU admissions and three separate models of NICU LOS. By building multiple models to analyze the impact of the technological intervention, we were able to provide robust estimates of the effect of the new technology. Across models, we found both consistency in direction (improved outcomes) and statistical significance. By controlling for maternal comorbidities, the true technological effect was isolated. A significant strength of this study was the use of the BSTS model to infer counterfactuals and be able to estimate the causal impact, which is usually difficult to do in observational studies of this nature. With the technological intervention, the CareFirst CHPDC care managers were able to use the web application to prioritize workflow and identify priority enrollees, specifically, pregnant enrollees with the greatest number of risk factors to perform a targeted approach. Through individualized, enrollee-centered management of pregnancies early in gestation, the NICU admissions rate and LOS both declined. This new technology may be applied not only to other health plans with a different enrollee population but also to other neonatal and maternal outcomes besides NICU admissions and LOS such as low birth weight and preterm birth.

The limitation of the current work is its retrospective nature. We controlled for birth volume, which captured any variations that may have arisen from increases in caseload due to either the technological implementation or structural changes to their membership. An additional limitation is that we did not consider reductions in stillbirths and pregnancy terminations using our new technology. Future areas of research include analyzing preterm birth, C-sections, stillbirths, and pregnancy terminations utilizing our new technology. Another limitation of our study is that we did not control for changes in obstetrics practice. We did control for time effects by including months and controlled for a history of PROM, preterm birth, and C-sections in our models.

It is possible that the risk scoring model in the technological intervention will miss a truly risky person, as it depends on the accuracy of the claims data that is received. For example, if a certain condition was not documented or not discovered by the physician at the time of the visit, the person may have a lower risk score than they should have. This would delay the appropriate intervention for that particular enrollee and potentially result in a worse outcome. However, one advantage of the technological intervention was that the risk scores were updated in real-time as new information was received, so if the condition was identified in a subsequent visit, the patient’s risk score was appropriately adjusted.

## Conclusions

Traditional care management tools and processes used before our new technology were manual and ad hoc and resulted in delayed pregnancy identification and proper risk assessment, which would result in worse NICU outcomes. Our new technology equips care managers with better tools for more timely pregnancy identification and more accurate risk stratification, which can lead to significant improvements in neonatal health outcomes as reflected by a reduction in NICU admissions and LOS. 

In our study, we saw improvements of as much as 29.2% lower odds of NICU admissions, 7.5%-58.5% fewer days in the NICU, as well as an average reduction of 48 NICU admissions and 528 NICU days per year. Implementing the new technology will result in healthier neonates and significantly reduce healthcare costs associated with NICU utilization. Future studies will focus on neonates who are receiving the biggest impacts from the new technology such as preterm or low birth weight neonates.
